# Gemtuzumab Ozogamicin and Stem Cell Mobilization for Autologous Stem Cell Transplantation in Favorable Risk Acute Myeloid Leukemia

**DOI:** 10.3390/biomedicines12071616

**Published:** 2024-07-19

**Authors:** Danaë Martinez Flores, Dilara Akhoundova, Katja Seipel, Myriam Legros, Marie-Noelle Kronig, Michael Daskalakis, Ulrike Bacher, Thomas Pabst

**Affiliations:** 1 Department of Medical Oncology, Inselspital, Bern University Hospital, University of Bern, 3010 Berne, Switzerland; danae.martinez@students.unibe.ch (D.M.F.); dilara.akhoundovasanoyan@insel.ch (D.A.); katja.seipel@insel.ch (K.S.); marie-noelle.kronig@insel.ch (M.-N.K.); 2 Department of Clinical Chemistry and Center for Laboratory Medicine, Inselspital, Bern University Hospital, University of Bern, 3010 Berne, Switzerland; myriam.legros@insel.ch; 3 Department of Hematology and Central Hematology Laboratory, Inselspital, Bern University Hospital, University of Bern, 3010 Berne, Switzerland; michael.daskalakis@insel.ch (M.D.); veraulrike.bacher@insel.ch (U.B.)

**Keywords:** acute myeloid leukemia (AML), gemtuzumab ozogamicin (GO), autologous stem cell transplantation (ASCT), peripheral blood stem cells (PBSC), stem cell mobilization

## Abstract

Gemtuzumab ozogamicin (GO), a CD33-targeting antibody drug conjugate, previously showed longer relapse-free survival when combined with induction chemotherapy in patients with favorable-risk acute myeloid leukemia (AML). In this patient population, characterized by lower relapse risk as compared to other ELN risk groups, autologous stem cell transplantation (ASCT) can be used as consolidation strategy. However, there are limited data on the impact of GO on the peripheral blood stem cell (PBSC) mobilization potential. We therefore retrospectively analyzed data from 54 AML patients with favorable-risk AML treated with (*n* = 17) or without (*n* = 37) GO during induction treatment. We observed no significant differences in the PBSC mobilization rate between patients treated with vs. without GO. The mobilization success in a first attempt directly following cycle 2 was 65% vs. 70% (*p* = 0.92); and the mobilization success in a subsequent second attempt after hematologic recovery and repeated stimulation procedure was 24% vs. 19% (*p* = 0.56). No significant impact on treatment outcome in terms of EFS (*p* = 0.31) or OS (*p* = 0.99) was observed. Thus, our results suggest that the addition of GO to induction regimens does not negatively impact PBSC mobilization in favorable-risk AML patients. To our best knowledge, this is the first study comparing the stem cell mobilization potential in favorable-risk AML patients treated with vs. without GO.

## 1. Introduction

The therapeutic management of acute myeloid leukemia (AML) consists of an initial induction treatment aiming to achieve complete remission (CR), followed by a post-remission consolidation to prevent relapse [1]. In patients classified as European LeukemiaNet (ELN) favorable-risk, cytarabine- and anthracycline-based induction treatment according to the 7 + 3 regimen remains the standard of care [1,2]. Gemtuzumab ozogamicin (GO), an antibody–drug conjugate composed of calicheamicin linked to a CD33-targeting monoclonal antibody, is Food and Drug Administration (FDA)-approved for adult patients with previously untreated CD33+ AML in combination with daunorubicin and cytarabine, based on the results of the Acute Leukemia French Association (ALFA)-0701 trial [3,4,5,6]. In this randomized open-label phase 3 study, patients aged 50–70 were randomized to receive induction treatment with cytarabine + daunorubicin with or without GO (3 mg/m^2^), administered on days 1, 4, and 7 during induction. Patients in the GO group showed a significantly longer event-free survival (median: 17.3 vs. 9.5 months; hazard ratio (HR): 0.66, *p* = 0.006) as compared to the control arm, with a greater efficacy observed in the favorable and intermediate ELN-risk groups. Prolonged time to platelet recovery compared to the control arm was the main side effect observed in the GO group [6,7]. These findings revealed that GO has a myelosuppressive effect [4] and might also potentially affect non-leukemic CD34+ stem cells.

In agreement with the ELN guidelines, favorable-risk AML patients are commonly consolidated with autologous stem cell transplantation (ASCT) [2,8,9], and granulocyte colony-stimulating factors (G-CSF) are used to mobilize CD34+ stem cells from the bone marrow. This graft stem cell source is referred to as peripheral blood stem cells (PBSC) [10]. The main goal of our single-center retrospective study was to assess whether the addition of GO to AML induction regimens is associated with a poorer PBSC mobilization in favorable-risk AML patients.

## 2. Materials and Methods

### 2.1. Patient Selection

In this study, we analyzed data from all consecutive patients with previously untreated de novo or secondary favorable-risk AML, treated at the University Hospital of Bern, Switzerland between 2013 and 2023. The following inclusion criteria were used: de novo or secondary AML, ELN favorable-risk, patients who received two induction cycles with cytarabine + anthracycline or cytarabine alone and at least one mobilization attempt. We elected 54 patients and separated them into the GO group (*n =* 17) and the control group (*n =* 37).

### 2.2. Procedures

All patients received two cycles of induction treatment consisting of cytarabine alone for seven days or in combination with an anthracycline (idarubicin/daunorubicin). For patients receiving the combination, induction cycle 1 consisted of cytarabine 200 mg/m^2^ (d1–7), idarubicin 12 mg/m^2^ (d1–3), +/− GO 3 mg/m^2^ (with capping at 5 mg total/d; at d 1, 4 and 7). Induction cycle 2 comprised cytarabine 1000 mg/m^2^ q12 h (d1–6), daunorubicin 65 mg/m^2^ (d1, 3 and 5), +/− GO 3 mg/m^2^ (with capping at 5 mg total/d; at d 1, 4 and 7). One patient in the GO group additionally received venetoclax and one patient in the control group, cladribine. Throughout the hematological recovery phase of the second cycle, patients were stimulated with G-CSF for peripheral CD34+ mobilization up to and including the first day of apheresis. Patients not achieving sufficiently high levels of peripheral CD34+ cells underwent a second mobilization attempt with the addition of vinorelbine followed by G-CSF. In patients treated after 2017, minimal residual disease (MRD) status was determined before apheresis in bone marrow aspirate. These data were not available for all patients treated before 2017, as they were not determined routinely. If apheresis was successfully performed, patients proceeded to ASCT following high-dose chemotherapy with treosulfan and melphalan, busulfan and melphalan, or busulfan and cyclophosphamide.

### 2.3. Study Design

The primary endpoint of this study was the feasibility of successful collection of peripheral CD34+ cells following the administration of GO during induction treatment. Therefore, we compared the percentage of patients who were able to successfully complete stem cell collection in the GO vs. in the control group. We defined the G-CSF mobilization following the second induction cycle as the first mobilization attempt. Mobilization failure on the first attempt was considered when patients did not achieve sufficient levels of peripheral CD34+ cells for subsequent apheresis. If possible, a second mobilization attempt was performed, and like the first one, if harvesting was not reliable, we classified it as mobilization failure on second attempt.

### 2.4. Statistics

**Figure 1:**
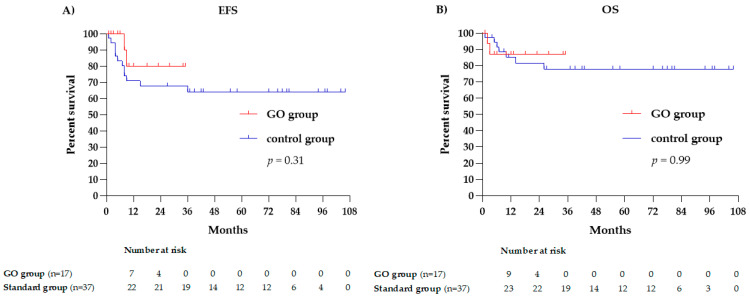
Kaplan—Meier curves illustrating (**A**) event-free survival of GO vs. control group; (**B**) overall survival of GO group and control group.

Event-free survival (EFS) was defined as the time from the start of the second cycle until the first relapse or last follow-up. Overall survival (OS) had the same starting point as EFS and ending point was death of any cause or last follow-up. Since CR could be first detected after the first induction cycle, we selected the date of the second induction cycle as the starting timepoint for EFS and OS calculation. We censored patients receiving allogenic stem cell transplantation (allo-SCT) after relapse on the date the allo-SCT was performed. Data were locked on 29 December 2023. EFS and OS analyses were calculated by using the log-rank (Mantel–Cox) test and visualized in Kaplan–Meier curves (Figure 1). Our survival analysis corresponds to an intention-to treat analysis, since we included all patients having at least one mobilization attempt regardless of whether it was successful and lead to ASCT as post-remission therapy or not.

Data on continuous variables we compared by using the Mann–Whitney U test, whereas we applied the Chi^2^ test with Yate’s correction for continuity for categorical variables. A *p*-value *≤* 0.05 was considered as significant. All statistical analysis were performed with GraphPad Prism^®^ Version 8.0.1.

## 3. Results

A total of 54 favorable-risk AML patients were included in the study: 17 patients within the GO group and 37 patients in the control group. Patient basal characteristics, including clinical presentation (primary vs. secondary AML), FAB subtype and molecular abnormalities, were evenly distributed between both patient cohorts. Patient basal characteristics are shown in Table 1. All 17 patients in the GO group received GO during induction treatment, ten of them during one cycle and seven of them in both cycles.

### 3.1. Mobillization

At the start of mobilization, white blood cell counts in the GO group were lower compared to the control group (median: 0.17 G/L vs. 0.60 G/L, *p* = 0.02). The median duration of G-CSF administration was longer in the GO group (7 days vs. 4 days, *p* = 0.08). A total of 11 patients in the GO group and 26 patients in the control group were successfully harvested during a first mobilization attempt directly following hematologic recovery from cycle 2 (65% vs. 70%, *p* = 0.92). Mobilization during a second attempt—with a repeated stimulation procedure—was successful in an additional 4 (24%) vs. 7 (19%) patients, *p* = 0.56. In the GO group, 5 out of the 6 patients with first-attempt mobilization failure underwent a second mobilization attempt, whereby 1 (6%) patient did not achieve adequate levels of CD34+ cells for subsequent apheresis in the second attempt. In the control group, 9 patients out of the 11 with mobilization failure during the first attempt underwent a second mobilization attempt, whereas 7 of them were successfully harvested and 2 (5%) patients experienced mobilization failure of the second attempt. Overall, no statistically significant differences were observed.

### 3.2. Apheresis and ASCT

MRD status before apheresis was available for all patients from the GO group and 10/37 patients from the control group. Out of 17 patients in the GO group, 13 were MRD negative, compared to 8 out of 10 patients with available MRD data in the control group. The amount of circulating CD34+ cells was significantly lower in the GO group than in the control group (25.1 × 10^6^/L vs. 46.6 × 10^6^/L, *p* = 0.04) at the day of apheresis. The difference in the amount of collected CD34+ cells between the two groups was not statistically significant (5.23 × 10^6^/kg b.w. vs. 9.02 × 10^6^/kg b.w., *p* = 0.07). Fifteen (88%) patients in the GO group and 33 (89%) in the control group successfully mobilized and harvested, when considering both mobilization attempts. No significant differences were observed in the median quantity of transplanted CD34+ cells (3.16 × 10^6^/kg b.w. vs. 4.31 × 10^6^/kg b.w., *p* = 0.06). The median time to neutrophil recovery (≥0.5 G/L) was 12 days in both groups, whereas the median time to platelet recovery (>20 G/L) was longer in the GO group (35 days vs. 22 days, *p* = 0.04). Patient characteristics regarding mobilization, apheresis, and ASCT metrics are shown in Table 2.

### 3.3. Clinical Outcome

Overall, 48 out of 54 (89%) patients received ASCT as post-remission therapy. Of these, 4 patients received high-dose cytarabine, 1 in the GO group and 3 in the control group. One patient from each group did not receive post-remission therapy. Overall, 14 out of 54 (26%) patients analyzed experienced relapse. Relapse and mortality rates were numerically lower in the GO group than in the control group: 2/17 (12%) vs. 12/37 (32%), *p* = 0.2 and 3/17 vs. 10/37 (18% vs. 27%, *p* = 0.68), respectively. However, these differences were not statistically significant (Table 3). Neither the EFS nor the OS was significantly longer between both patient groups (*p* = 0.31 and *p* = 0.99, respectively). Four patients were censored in the survival curves on the day of their allo-SCT.

## 4. Discussion

In this study, we analyzed whether the addition of GO to induction chemotherapy in favorable-risk AML patients was associated with impaired stem cell mobilization outcomes. For this purpose, we retrospectively compared two AML patient cohorts, treated with or without GO during induction therapy. GO combined with chemotherapy currently constitutes a standard of care in the management of patients with newly diagnosed CD33-positive favorable-risk AML.

Several randomized trials have shown that GO, when added to induction chemotherapy, improves outcomes in this patient population [11,12]. The UK Medical Research Council (MRC) AML15 trial analyzed the effect of a single dose of GO (3 mg/m^2^) administrated on day one during induction therapy. In this trial, 1113 patients aged 15–71 with previously untreated de novo or secondary AML received two induction cycles of chemotherapy, with one of the following regimens: daunorubicin and cytarabine; daunorubicin, cytarabine and etoposide; or fludarabine, cytarabine, and idarubicin. Patients were randomly assigned to receive or not receive GO in addition to induction chemotherapy. Although no difference was found regarding relapse rate, relapse-free survival or OS between patient groups, a prespecified analysis by cytogenetics showed improved OS in favorable-risk patients [11].

Similarly, the UK National Cancer Research Institute (NCRI) AML16 randomized trial analyzed the benefit of a single dose of GO (3 mg/m^2^) administered on day one of the first induction cycle in patients aged 51–84. Induction regimens used in patients with de-novo AML, secondary AML or high-risk myelodysplastic syndrome were either daunorubicin combined with cytarabine or with clofarabine, with (*n* = 559) or without (*n* = 556) additional administration of GO. The trial demonstrated a lower 3-year cumulative incidence of relapse (68% vs. 76%, *p* = 0.007) and a better 3-year overall survival rate (25% vs. 20%, *p* = 0.05) in the GO group compared to the control arm.

A meta-analysis including data from both, the MRC AML15 and the NCRI AML16 trials, was conducted which compiled information regarding the GO dose, application time and induction regimen from both trials. Data analysis from a total of 2228 patients with a median age of 61 confirmed a significantly reduced risk of relapse (*p* = 0.002) and improved survival (*p* = 0.02) when adding GO to the induction treatment [12]. The MRC AML15 trial as well as the NCRI AML16 trial were part of another meta-analysis published in 2014 including five randomized controlled trials, which assessed efficacy of GO added to first course of induction treatment. The meta-analysis combined data from more than 3300 patients and showed a benefit in relapse risk (OR 0.81, 0.73–0.90; *p* = 0.0001) and OS at 5 years (OR 0.90, 0.82–0.98; *p* = 0.01) [13].

The ALFA-0701 trial showed the benefit of adding GO in NPM1 mutated patients [7]. Moreover, the predictive value of NPM1 was analyzed in the German–Austrian Acute Myeloid Leukemia Study Group (AMLSG) 09-09 phase III study, which compared patients with NPM1-mutated AML receiving two courses of induction therapy consisting of idarubicin, etoposide and all-trans-retinoic acid with or without GO, respectively. GO was administered as a single dose on day one (3 mg/m^2^) during induction and the first consolidation cycle. The study failed to demonstrate an improvement in EFS or OS in the GO group; however, addition of GO led to a lower relapse rate. Patients receiving GO experienced prolonged thrombocytopenia (median platelet recovery following the second induction cycle: 25 days in the GO arm vs. 20 days in the control arm; *p* ≤ 0.001) [14]. Prolonged treatment induced thrombocytopenia and neutropenia were also reported in the ALFA 0701 trial [7]. Our observation of lower WBC levels on the first day of G-CSF stimulation (median 0.17 G/L vs. median 0.60 G/L, *p* = 0.02) in the GO group would be in line with these findings.

A potential impact on hematological recovery as well as the myelosuppressive effects of GO is also suggested by our findings of significantly lower circulating CD34+ cells at apheresis day (25.1 × 10^6^/L vs. 46.6 × 10^6^/L, p = 0.04). Moreover, since time of platelet recovery to >20 G/L after ASCT was longer in the GO group compared to the control group (35 days vs. 22 days, *p* = 0.04), our data suggest that the myelosuppressive effect of GO might persist after HDCT and ASCT. In previous work, we showed that patients mobilizing more than 60,000 CD34+ cells/mL on apheresis day had significantly shorter OS (*p* = 0.0274) and time to progression (*p* = 0.0014) and a higher relapse rate (*p* = 0.0177) [15]. Furthermore, delayed hematological recovery, defined as recovery of neutrophils (>1 × 10^9^/L) and platelets (>20 × 10^9^/L) occurring 20 days or later after ASCT, has been associated with improved OS and time to progression (TTP) (OS, neutrophils: *p* =< 0.0001 and platelets: *p* =< 0.0062; TTP, neutrophils: *p* =< 0.0003 and platelets: *p* =< 0.0125) [16]. In the current study, we did not observe differences regarding EFS or OS between the GO and the control groups; however, the limited sample size must be acknowledged.

In summary, our data showed no significant differences between the GO and the control groups with regards to the success of peripheral stem cell mobilization. However, patients receiving GO required longer G-CSF stimulation. These findings are in contrast to the results of a retrospective analysis of 20 patients being treated with fractionated doses of GO and the 7 + 3 regimen followed by 1–2 cycles of consolidation with high-dose cytarabine alone or in combination with daunorubicin and GO. Only 11 out of 20 patients mobilized successfully reaching a predefined threshold of 20 CD34+ cells/μL [17]. The retrospective single-center design of our study, the limited sample size and the short follow-up time, constitute potential limitations of our study.

## 5. Conclusions

Our study suggests that the use of GO during AML induction therapy does not have a negative impact on PBSC mobilization potential. To our best knowledge, this is the first study comparing stem cell mobilization metrics in favorable-risk AML patients treated with intensive induction therapy with vs. without GO. No differences in clinical outcomes for EFS or OS were detected. In conclusion, our findings indicate that the administration of GO during induction treatment should not limit the use of ASCT as consolidation strategy.

## Figures and Tables

**Table 1:** Clinical characteristics at diagnosis.

	GO Group (*n* = 17)	Control Group (*n* = 37)	All Patients (*n* = 54)	*p*
Median age at diagnosis, years (range)	52 (17–74)	54 (23–73)	53 (17–74)	0.72
Females, *n* (%)	5 (29)	22 (59)	27 (50)	0.08
Males, *n* (%)	12 (71)	15 (41)	27 (50)	
**Peripheral Blood Parameters**				
WBC ^1^ G/L median (range)	36 (1.4–214.6)	9.32 (1.54–202)	15.9 (1.4–214.6)	0.07
Platelets G/L, median (range)	53 (4–130)	76 (8–246)	71.5 (4–246)	0.34
Hemoglobin g/L, median (range)	83 (56–126)	82 (38–135)	82.5 (38–135)	0.80
LDH ^2^ U/L, median (range)	634 (162–2188)	716 (167–2086)	710 (162–2188)	0.76
Peripheral blasts %, median (range)	60 (0–98)	47 (0–95)	47 (0–98)	0.90
BM ^3^ blasts %, median (range)	80 (20–92.5)	85 (20–95)	80 (20–95)	0.34
**FAB ^4^ classification**				
M0, *n* (%)	1 (6)	0 (0)	1 (2)	0.69
M1, *n* (%)	5 (29)	8 (22)	13 (24)	0.70
M2, *n* (%)	3 (18)	13 (35)	16 (30)	0.32
M4, *n* (%)	6 (35)	14 (38)	20 (37)	0.90
M5, *n* (%)	2 (12)	2 (5)	4 (7)	0.79
**AML type**				
De novo AML, *n* (%)	16 (94)	34 (92)	50 (93)	0.79
Therapy related, *n* (%)	1 (6)	3 (8)	4 (7)	
**WHO recurrent abnormalities ^5^**				
*RUNX1-RUNX1T1*, *n* (%)	2 (12)	5 (14)	7 (13)	0.80
*CBFB-MYH11*, *n* (%)	3 (18)	6 (16)	9 (17)	0.79
*CEBPA*mut, *n* (%)	3 (18)	5 (14)	8 (15)	0.99
*NPM1*wt with *FLT3*-ITD, *n* (%)	0 (0)	1 (3)	1 (2)	0.69
*NPM1*mut with *FLT3*wt, *n* (%)	10 (59)	22 (59)	32 (59)	0.80

^1^ WBC: white blood cells; ^2^ LDH: lactat dehydrogenase; ^3^ BM: bone marrow; ^4^ FAB: French–American–British classification; ^5^ WHO: World Health Organization.

**Table 2:** Patient characteristics at mobilization, apheresis and ASCT.

	GO Group (*n* = 17)	Control Group (*n* = 37)	All Patients (*n* = 54)	*p*
Mobilization				
Day of mobilization start ^1^, median (range)	21 (10–27)	20 (10–28)	21 (10–28)	0.61
WBC ^2^ at day 1 of G-CSF ^3^ G/L, median (range)	0.17 (0.02–8.28)	0.60 (0–7.02)	0.48 (0–8.28)	0.02
Number of days G-CSF ^3,4^ stimulation, median (range)	7 (2–29)	4 (2–17)	6 (2–29)	0.08
Mobilization success/failure in first attempt, *n* (%)	11 (65)/6 (35)	26 (70)/11 (30)	37 (69)/17 (31)	0.92
Day of mobilization end ^1^ if failed, median (range)	29 (28–33)	30 (23–41)	30 (28–41)	0.67
WBC ^2^ at stop mobilization G/L, median (range)	7.14 (1.44–11.4)	7.35 (1.21–23.1)	7.35 (1.21–23.1)	0.88
CD34+ at stop mobilization ×10^6^/L, median (range)	0.7 (0.1–8.53)	1.1 (0–6.1)	1.1 (0–8.53)	0.79
Second mobilization attempt ^5^, *n* (%)	5 (29)	9 (24)	14 (26)	0.56
Mobilization success/failure in second attempt, *n* (%)	4 (24)/1 (6)	7 (19)/2 (5)	11 (20)/3 (6)	0.56
Apheresis				
Single day of apheresis/multiple days of apheresis, *n* (%)	14 (82)/1 (6)	30 (81)/3 (8)	44 (82)/4 (7)	0.78
WBC ^2^ at apheresis day G/L, median (range)	23.9 (7.11–63.8)	20 (3.14–63.6)	22.79 (3.14–63.8)	0.91
CD34+ at apheresis day ×10^6^/L, median (range)	25.1 (3.1–643.8)	46.6 (3–620.7)	38.35 (3–643.8)	0.04
Collected CD34+ cells ×10^6^/kg b.w. ^6^, median (range)	5.23 (2.09–32.15)	9.02 (2.81–58)	7.67 (2.09–58)	0.07
ASCT ^7^				
Patients transplanted, *n* (%)	15 (88)	33 (89)	48 (89)	0.72
Median time from diagnosis to ASCT, months (range)	3 (3–4)	3 (2–13)	3 (2–13)	0.07
Transplanted CD34+ ×10^6^/kg b.w. ^6^, median (range)	3.16 (2.09–10.84)	4.31 (1.88–18.05)	3.78 (1.88–18.05)	0.06
Median follow-up, months (range)	12 (1–35)	42 (1–106)	27 (1–106)	0.001
Time to neutrophil count ≥ 0.5 G/L ^8,9^, days, median (range)	12 (10–21)	12 (10–19)	12 (10–21)	0.37
Time to platelets > 20G/L ^8,10^, days, median (range)	35 (19–182)	22 (11–137)	26 (11–182)	0.04
Hospitalization duration ^11^, days, median (range)	22 (18–37)	25 (19–101)	25 (18–101)	0.32

^1^ Since start of cycle 2 induction chemotherapy; ^2^ WBC: white blood cells; ^3^ G-CSF: granulocyte colony-stimulating-factor; ^4^ 1 pt. in the GO group did not receive Filgrastim but was stimulated with pegfilgrastim singularly, 1 pt. in the GO group was additionally treated 3 days with plerixavor; ^5^ 1 pt. in the GO group and 2 pt. in the control group had no second mobilization attempt; ^6^ b.w.: body weight; ^7^ ASCT: autologous stem cell transplantation; ^8^ since day of ASCT, in which day of transplantation is day 0; ^9^ 1 pt. in the go group did not achieve neutrophils > 0.5 G/L; ^10^ 2 pt. in the GO group and 1 pt. in the control group did not recover > 20 G/L; ^11^ for high dose chemotherapy followed by autologous stem cell transplantation.

**Table 3:** Outcomes.

	GO Group (*n* = 17)	Control Group (*n* = 37)	All Patients (*n* = 54)	*p*
Relapse, *n* (%)	2 (12)	12 (32)	14 (26)	0.20
Median interval to relapse ^1^, moths (range)	8.5 (8–9)	6 (1–36)	7.5 (1–36)	0.36
Death, *n* (%)	3 (18)	10 (27)	13 (24)	0.68
Median time to death ^1^, months (range)	3 (2–15)	11 (1–28)	10 (1–28)	0.35
Death due to progression, *n* (%)	3 (18)	8 (22)	11 (20)	0.94
Death due to other causes ^2^, *n* (%)	0 (0)	2 (5)	2 (4)	

^1^ Since start of cycle 2 induction chemotherapy; ^2^ biopsy-related complication (1 pt.), pneumonia with ARDS (1 pt.).

## Data Availability

The data presented in this study are available upon request from the corresponding author.
